# Supplementary material for: Bovine breed-specific augmented reference graphs facilitate accurate sequence read mapping and unbiased variant discovery
Source: Genome Biol. 2020 Jul 27;21:184. doi: 10.1186/s13059-020-02105-0 (PMC7385871; doi:10.1186/s13059-020-02105-0)
Supplement: Supplementary file 4 — Additional file 4. Review history [file 13059_2020_2105_MOESM4_ESM.docx]

Review History

**First round of review**

**Reviewer 1**

**Are you able to assess all statistics in the manuscript, including the appropriateness of statistical tests used?**

Yes, and I have assessed the statistics in my report.

**Comments to author:**

The manuscript, entitled "Bovine breed-specific augmented reference graphs facilitate accurate sequence read mapping and unbiased variant discovery" by Crysnanto et al. aimed to construct multiple variation-aware reference graphs for Bos taurus to improve SNP and indel calling. The authors mainly implemented the vg pipeline using the new cattle reference assembly, completed with Illumina short reads from 4 European cattle breeds. After testing multiple breed and filtering combinations, the authors evaluated the assembly impacts on SNP and short indel calling accuracy. They concluded that the MAF filtering between 0.01 to 0.1% worked best for their samples and a bovine pan-genome graph could be widely-applicable. All other conclusions seem to be mainly reconfirmations of earlier human results with no novel significance. Many technical renovations (such as incorporating existing cattle long read sequence, high quality assemblies, and structural variant information) are skipped thus reduced audience enthusiasm. I have the following

Major concerns:

1. Introduction is not balanced. Important papers like [18] and [22] were only mentioned briefly there. Although they were commented more in Discussion, author should introduce all human side developments, on which this work is heavily relying on, so the readers have an objective view about what have been done and what are the new contributions for this work.

2. It is important to quantitatively estimate the contributions from paralogous variations as compared to orthologous/allelic variations, i.e. structural variants. This sentence is quite superficial to me: "Incorrectly mapped reads that matched corresponding reference nucleotides primarily originated from repetitive and tandemly duplicated regions."

3. "However, the variation aware reference of our study does not facilitate to accurately map short reads to repetitive and duplicated regions." Again, this is because a similar effort like [18] was not conducted.

4. I do not understand what does "sequence variant" on Line 586 mean. Large sequence variant or structural variant? If so, this logic is very circumstantial.

5. Many of results are expected or already known, for example, Line 209-211, Line 271-274, and many other places in Discussion.

6. "Bias towards the reference allele was more pronounced at insertion than deletion polymorphisms." Of course, this is as expected because only insertions were represented in the reference sequence when they are incorporated in, while deletions are already detectable.

7. Line 598, multiple taurine (Angus, Highland) and indicine (Brahman) long-read, high-quality assemblies and their associated alleles/haplotypes and structural variant information are available, so a similar work like Li et al. [47] is possible.

8. Maybe a performance comparison between cattle vs. human pangenomes in terms of genetic divergence within species will be more interesting.

Minor things:

9. Page 3 Line 71: 2'700 to 2,700

10. Page 6 Figure 2C, color meanings?

11. Citation [53] page numbers seem wrong?

**Reviewer 2**

**Are you able to assess all statistics in the manuscript, including the appropriateness of statistical tests used?**

Yes, and I have assessed the statistics in my report.

**Comments to author:**

The authors share new reference pan-genomes and benchmark new strategies for aligning sequencing reads to cow genomes. The authors note the deficiency of the current reference genome, which matches the Hereford cow breed but lacks any of the genetic diversity of other breeds. In a thorough series of benchmarks, the authors show that pan genomes built from genotype data from the 1000 Bull Genomes Project, when used in conjunction with the vg graph aligner, provide markedly better read alignment accuracy compared to linear genomes and linear aligners like BWA-MEM. They show that breed-specific pan genomes help slightly more than a global cross-breed pan genome.

While the results are not surprising, and follow the kinds of results seen in prior studies in human (FORGe), it is nonetheless a is compelling story, and the benchmarking is more thorough than many similar studies. The authors have effectively zoomed in on the metrics where the differences between the reference representations are most pronounced.

The writing is very clear.

The data sharing is commendable.

Major comments:

The manuscript needs to be clearer about which experiments are using a single chromosome and which are using the entire cow genome. I have listed specific places where this was not clear to me (without cross-referencing to other parts of the paper) under Minor Comments below.

The FORGe study used alignment to a "personalized" reference to establish an upper bound on how well an alignment method could do. This study can and should do the same. This conveys much more to the reader than if the upper bound is unstated.

Minor comments:

It was not clear what significance to ascribe to the edge-to-node ratio. The text says: "the edge-to-node ratio increased when variants were added to the graph (Pearson R=0.99), reflecting that the number of alternative paths increases disproportionately to the number of variants in the graph." Is this not obvious? A linear genome is essentially 1 node and 0 edges. A genome with one SNV in the middle will be 4 nodes and 4 edges. Generally speaking, it seems clear that adding N more nodes should add more than N more edges. Is the edge-to-node ratio really saying something beyond the number of nodes per se? It seems that the "number of alternative paths" is something that should increase exponentially in the number of nodes or edges, but edge-to-node ratio is not increasing at that rate.

Figure 2 legend should state (a) that it's just chromosome 25, and (b) the fact that the input data are simulated.

Figure 3 legend and the beginning of section titled "Mapping to breed-specific augmented genome graphs" should both specify whether the focus is on a single chromosome or the entire genome.

The notion of a "perfect" alignment should be clarified. Does it mean no mismatches or gaps? No soft clipping? Highest possible mapping quality?

In the Discussion, the authors write that "Grytten et al. [34] observed that an adjusted parameter setting of BWA mem improves linear mapping. Because we compared graph-based mapping to the most-widely used linear mapping approach, we did not tune BWA mem." I did not understand this point. It seems to me that the point made by Grytten et al -- that the linear aligner can be easily tuned so as to make up a lot of the difference with the graph aligner but without actually using a graph -- is quite relevant. Readers will come away not knowing whether tuned BWA-MEM might not have made up the difference.

I was confused by some uses of the term "private." I expected it to describe variants appearing in exactly one breed, but it seems to sometimes be used to refer to variants that are simply absent from one breed. For example: "The bovine linear reference sequence does neither contain allelic variation nor nucleotides that are private to breeds other than Hereford."

The Discussion is rather long, choosing to rehash all of the results rather than summarizing or synthesizing them.

**Authors Response**

**Point-by-point responses to the reviewers’ comments:**

Reviewer #1: The manuscript, entitled "Bovine breed-specific augmented reference graphs facilitate accurate sequence read mapping and unbiased variant discovery" by Crysnanto et al. aimed to construct multiple variation-aware reference graphs for Bos taurus to improve SNP and indel calling. The authors mainly implemented the vg pipeline using the new cattle reference assembly, completed with Illumina short reads from 4 European cattle breeds. After testing multiple breed and filtering combinations, the authors evaluated the assembly impacts on SNP and short indel calling accuracy. They concluded that the MAF filtering between 0.01 to 0.1% worked best for their samples and a bovine pan-genome graph could be widely-applicable. All other conclusions seem to be mainly reconfirmations of earlier human results with no novel significance. Many technical renovations (such as incorporating existing cattle long read sequence, high quality assemblies, and structural variant information) are skipped thus reduced audience enthusiasm.

*Author: Thank you for your valuable comments on our manuscript.*

I have the following

Major concerns:

Reviewer1_1: Introduction is not balanced. Important papers like [18] and [22] were only mentioned briefly there. Although they were commented more in Discussion, author should introduce all human side developments, on which this work is heavily relying on, so the readers have an objective view about what have been done and what are the new contributions for this work.

*Author: We have now added relevant information to the introduction section and clarified the contributions of our work (l. 96-107).*

Reviewer1_2: It is important to quantitatively estimate the contributions from paralogous variations as compared to orthologous/allelic variations, i.e. structural variants. This sentence is quite superficial to me: "Incorrectly mapped reads that matched corresponding reference nucleotides primarily originated from repetitive and tandemly duplicated regions."

*Author: We analyzed the origin of the unmappable reads using data from gene annotation and RepeatMasker. We present the mapping statistics specific to the different sequence features in the revised manuscript and in a newly compiled additional figure (l. 435 – 444, Figure S12). We now state (“Similar to findings in human genome graphs [18, 25], bovine variation-aware references did not improve the mapping of short reads that originate from low-complexity regions.”). We are not aware that long-read sequencing or assembly data are available for the breeds considered in our analysis. This is a limitation of our study that is mentioned explicitly in the revised version (l. 715, l. 740, l. 746). Moreover, we added data on structural variants detected from short sequencing reads (l. 446, Supplementary note 3) and discussed the limitations (l. 748).*

Reviewer1_3: "However, the variation aware reference of our study does not facilitate to accurately map short reads to repetitive and duplicated regions." Again, this is because a similar effort like [18] was not conducted.

*Author: We were not sure to what analysis of FORGe the reviewer referred to. Thus, we performed two additional analyses and revised the paper accordingly. First, we classified the origin of the reads (see comment above, Figure S12). Second, we ran the methods implemented in FORGe to prioritize variants (l. 205 – 215, Supplementary note 1). We observed that our ranking method yields the same mapping accuracy as Pop Cov. Other methods did not lead to further accuracy improvements. Thus, variant ranking using FORGe will not solve the low mapping accuracy of short-reads in repetitive regions.*

Reviewer1_4: I do not understand what does "sequence variant" on Line 586 mean. Large sequence variant or structural variant? If so, this logic is very circumstantial.

*Author: We clarified that sequence variants refers to SNPs and small Indels (Line 711).*

Reviewer1_5: Many of results are expected or already known, for example, Line 209-211, Line 271-274, and many other places in Discussion.

*Author: We agree that our results broadly confirm findings from human populations. We feel that this is a broadly relevant and important finding because the genetic diversity and allele frequency distribution differs considerably between cattle and human populations. We now added an entire section to compare bovine and human graphs (see comment to the Editor above) and highlight the differences between the species in the discussion section. Moreover, we revised the entire discussion section in order to avoid repetitions and better highlight our own contribution because this was also suggested by Reviewer #2.*

Reviewer1_6: "Bias towards the reference allele was more pronounced at insertion than deletion polymorphisms." Of course, this is as expected because only insertions were represented in the reference sequence when they are incorporated in, while deletions are already detectable.

*Author: We clarified that this is an expected finding (l. 542)*

Reviewer1_7: Line 598, multiple taurine (Angus, Highland) and indicine (Brahman) long-read, high-quality assemblies and their associated alleles/haplotypes and structural variant information are available, so a similar work like Li et al. [47] is possible.

*Author: We struggle a bit with this comment. Our analyses are based on the recently released cattle reference genome ARS-UCD1.2 which is of excellent quality (N50>25 MB) (Rosen et al. 2020). Breed-specific assemblies might serve as tailored backbones for breed-specific augmented genome graphs because they might contain nucleotides that are missing in the current assembly. We mention this option in the discussion (l. 743). We are aware that reference-quality assemblies are available for the Angus, Brahman and Scottish Highland breeds. However, these beef breeds are genetically distinct from the dairy and dual-purpose breeds of our study. Long-read sequencing data is to the best of our knowledge not available for the cattle breeds considered in our study. In order to convey this information more explicitly, we mention this limitation now in the discussion section (l. 715, l. 740, l. 746). The construction of a pangenome-graph that unifies multiple genome assemblies is out of scope of the present manuscript. However, we mention this possibility in the discussion (l. 752) and added a new reference to the minigraph pre-print.*

Reviewer1_8: Maybe a performance comparison between cattle vs. human pangenomes in terms of genetic divergence within species will be more interesting.

*Author: We added an entire section to compare bovine and human graphs (see comment to the Editor above) in the revised manuscript and highlight the differences between the species in the discussion section.*

Minor things:

Reviewer1_9: Page 3 Line 71: 2'700 to 2,700

*Author: Thanks for catching this mistake. We have corrected the corresponding value.*

Reviewer1_10: Page 6 Figure 2C, color meanings?

*Author: We added the meanings of the color in the legend (Figure 2f in the revised manuscript).*

Reviewer1_11: Citation [53] page numbers seem wrong?

*Author: We removed the page numbers from the citation, because it is an online-only article.*

Reviewer#2

Reviewer #2: The authors share new reference pan-genomes and benchmark new strategies for aligning sequencing reads to cow genomes. The authors note the deficiency of the current reference genome, which matches the Hereford cow breed but lacks any of the genetic diversity of other breeds. In a thorough series of benchmarks, the authors show that pan genomes built from genotype data from the 1000 Bull Genomes Project, when used in conjunction with the vg graph aligner, provide markedly better read alignment accuracy compared to linear genomes and linear aligners like BWA-MEM. They show that breed-specific pan genomes help slightly more than a global cross-breed pan genome.

While the results are not surprising, and follow the kinds of results seen in prior studies in human (FORGe), it is nonetheless a is compelling story, and the benchmarking is more thorough than many similar studies. The authors have effectively zoomed in on the metrics where the differences between the reference representations are most pronounced.

The writing is very clear.

The data sharing is commendable.

*Author: Thank you for your valuable and encouraging comments on our manuscript.*

Major comments:

Reviewer#2_1: The manuscript needs to be clearer about which experiments are using a single chromosome and which are using the entire cow genome. I have listed specific places where this was not clear to me (without cross-referencing to other parts of the paper) under Minor Comments below.

*Author: We clarified the use of either one chromosome or the entire genome at multiple instances in the revised manuscript.*

Reviewer#2_2: The FORGe study used alignment to a "personalized" reference to establish an upper bound on how well an alignment method could do. This study can and should do the same. This conveys much more to the reader than if the upper bound is unstated.

*Author: We construct a personalized genome graph that contains only the haplotypes of the animals used for read simulation. This analysis was added to the main part of the manuscript (l. 361 f.). We adjusted Figure 4 and all corresponding additional Figures (S7, S10, S11) accordingly.*

Minor comments:

Reviewer#2_3: It was not clear what significance to ascribe to the edge-to-node ratio. The text says: "the edge-to-node ratio increased when variants were added to the graph (Pearson R=0.99), reflecting that the number of alternative paths increases disproportionately to the number of variants in the graph." Is this not obvious? A linear genome is essentially 1 node and 0 edges. A genome with one SNV in the middle will be 4 nodes and 4 edges. Generally speaking, it seems clear that adding N more nodes should add more than N more edges. Is the edge-to-node ratio really saying something beyond the number of nodes per se? It seems that the "number of alternative paths" is something that should increase exponentially in the number of nodes or edges, but edge-to-node ratio is not increasing at that rate.

*Author: Apologies for the confusion. Using the vg toolkit, the maximum number of bases in a single node is 32, i.e., the vg-based graph representation of the linear genome != 1 node and 0 edges. The graph-based representation of the empty bta25 contains 1,323,451 nodes and 1,323,450 edges (l. 163). Adding a single SNVs to the linear graph adds 3 nodes (reference node, alternate variant node, and split node from the original node) and 4 edges (incoming and outgoing edges of the reference and alternate nodes). We clarified the entire section (l. 153) and added an additional figure S1.*

Reviewer#2_4: Figure 2 legend should state (a) that it's just chromosome 25, and (b) the fact that the input data are simulated.

*Author: Done.*

Reviewer#2_5: Figure 3 legend and the beginning of section titled "Mapping to breed-specific augmented genome graphs" should both specify whether the focus is on a single chromosome or the entire genome.

*Author: Done.*

Reviewer#2_6: The notion of a "perfect" alignment should be clarified. Does it mean no mismatches or gaps? No soft clipping?

*Author: We clarified the notion in the revised manuscript (l. 494, l.980)*

Reviewer#2_7: In the Discussion, the authors write that "Grytten et al. [34] observed that an adjusted parameter setting of BWA mem improves linear mapping. Because we compared graph-based mapping to the most-widely used linear mapping approach, we did not tune BWA mem." I did not understand this point. It seems to me that the point made by Grytten et al -- that the linear aligner can be easily tuned so as to make up a lot of the difference with the graph aligner but without actually using a graph -- is quite relevant. Readers will come away not knowing whether tuned BWA-MEM might not have made up the difference.

*Author: We optimized the linear mapping approach accordingly and provide results that are based on the BWA-mem + Minimap2 approach proposed by Grytten et al., in the revised manuscript (l. 390-400, supplementary note 2). We found that tuning the linear approach increases mapping quality only slightly likely because the proportion of reads with variants is considerably larger in cattle than humans. We also added this information to the discussion (l. 625-630)*

Reviewer#2_8: I was confused by some uses of the term "private." I expected it to describe variants appearing in exactly one breed, but it seems to sometimes be used to refer to variants that are simply absent from one breed. For example: "The bovine linear reference sequence does neither contain allelic variation nor nucleotides that are private to breeds other than Hereford."

*Author: Thank you for this comment. We replaced all instances of “private” in the manuscript to be clearer.*

Reviewer#2_9: The Discussion is rather long, choosing to rehash all of the results rather than summarizing or synthesizing them.

*Author: We revised the entire discussion section and moved parts to the introduction (as suggested by reviewer#1) and highlighted the implications of our contribution.*

**Second round of review**

**Reviewer 2**

The revision is very thorough, addressing all my points and improving the study. It is strong and ready for publication.
